# Idiopathic Gingival Enlargement: A Case Report

**DOI:** 10.7759/cureus.65195

**Published:** 2024-07-23

**Authors:** Marde Shraddha Kashinath, Rohit Kumar Kendole

**Affiliations:** 1 Periodontics, ESIC Dental College, Kalaburagi, IND; 2 Oral and Maxillofacial Pathology, Community Health Center Rampura, Chitraduga, IND

**Keywords:** hereditary gingival fibromatosis, gingival hyperplasia, gingivoplasty, gingivectomy, idiopathic enlargement

## Abstract

Gingival enlargement (GE) is an increase in the size of the gingiva. It may be due to inflammation caused by extensive plaque accumulation, intake of drugs, systemic conditions like pregnancy and puberty, systemic diseases such as leukemia or Wegener’s granulomatosis, hereditary gingival fibromatosis, and neoplastic or false enlargement. Idiopathic GE is the massive increase in the size of the gingiva with an unknown etiology. It may have a hereditary basis, be linked to physical impairment, or begin with eruption of primary or permanent dentition. It is also referred as gingivomatosis, hereditary gingival fibromatosis, elephantiasis gingivae, gigantism of the gingiva, or congenital macrogingivae. The enlarged gingiva compromises oral hygiene maintenance, which secondarily adds to the inflammatory component of enlargement. Altogether, this exaggerates the existing condition. This type of extensively disfigured gingiva affects speech, mastication, and esthetics, causes halitosis, and disturbs the overall well-being of the individual. Surgical removal of the enlarged gingiva along with meticulous non-surgical means of plaque control is expected to provide a satisfactory functional and esthetic outcome. This case report presents a rare case of long-standing massive grade III GE extending up to the occlusal level in a 17-year-old systemically healthy, non-syndromic young female involving both arches, thereby posing a diagnostic dilemma. It was treated by gingivectomy using a conventional technique to facilitate precise incision, lower cost, and faster re-epithelialization. This was followed by gingivoplasty using electrocautery. The postoperative results of three months were satisfactory in terms of function and esthetics with uneventful healing. Further follow-up is ongoing for the same.

## Introduction

Gingival enlargement (GE) is an increase in the size of the gingiva. It may be due to inflammation caused by extensive plaque accumulation, intake of drugs known to affect gingival fibroblasts from a group of antiepileptics, antihypertensives or immunosuppressants, systemic conditions like pregnancy and puberty, systemic diseases such as leukemia or Wegener’s granulomatosis, hereditary gingival fibromatosis, and neoplastic or false enlargement. Idiopathic GE is also referred as gingivomatosis, hereditary gingival fibromatosis, elephantiasis gingivae, gigantism of the gingiva, or congenital macrogingivae. Hereditary gingival fibromatosis is a heterogeneous group of disorders characterized by an increase in the connective tissue element [1]. The etiology is poorly understood but can be attributed to extensive plaque accumulation caused by insufficient oral hygiene measures in malocclusion, hormonal changes, blood dyscrasias, genetic origin, or idiopathic [2]. This condition causes functional, esthetic, masticatory, and psychological disturbances.

This case report presents a rare case of long-standing massive grade III GE in a 17-year-old systemically healthy, non-syndromic young female involving both arches, thereby posing a diagnostic dilemma.

## Case presentation

A 17-year-old female patient reported to the outpatient department of periodontics with a chief complaint of swollen gums for 10 years. She had difficulty in closing her mouth, mastication, speech, and esthetic concerns. The patient’s parents reported her halitosis and psychosocial stigma due to the gingival condition. No significant medical or drug history was noted. No history of epilepsy, hypertension, hypertrichosis, ear pain, digital abnormalities, mental retardation, fever, or any other systemic illness was found.

**Figure 1:**
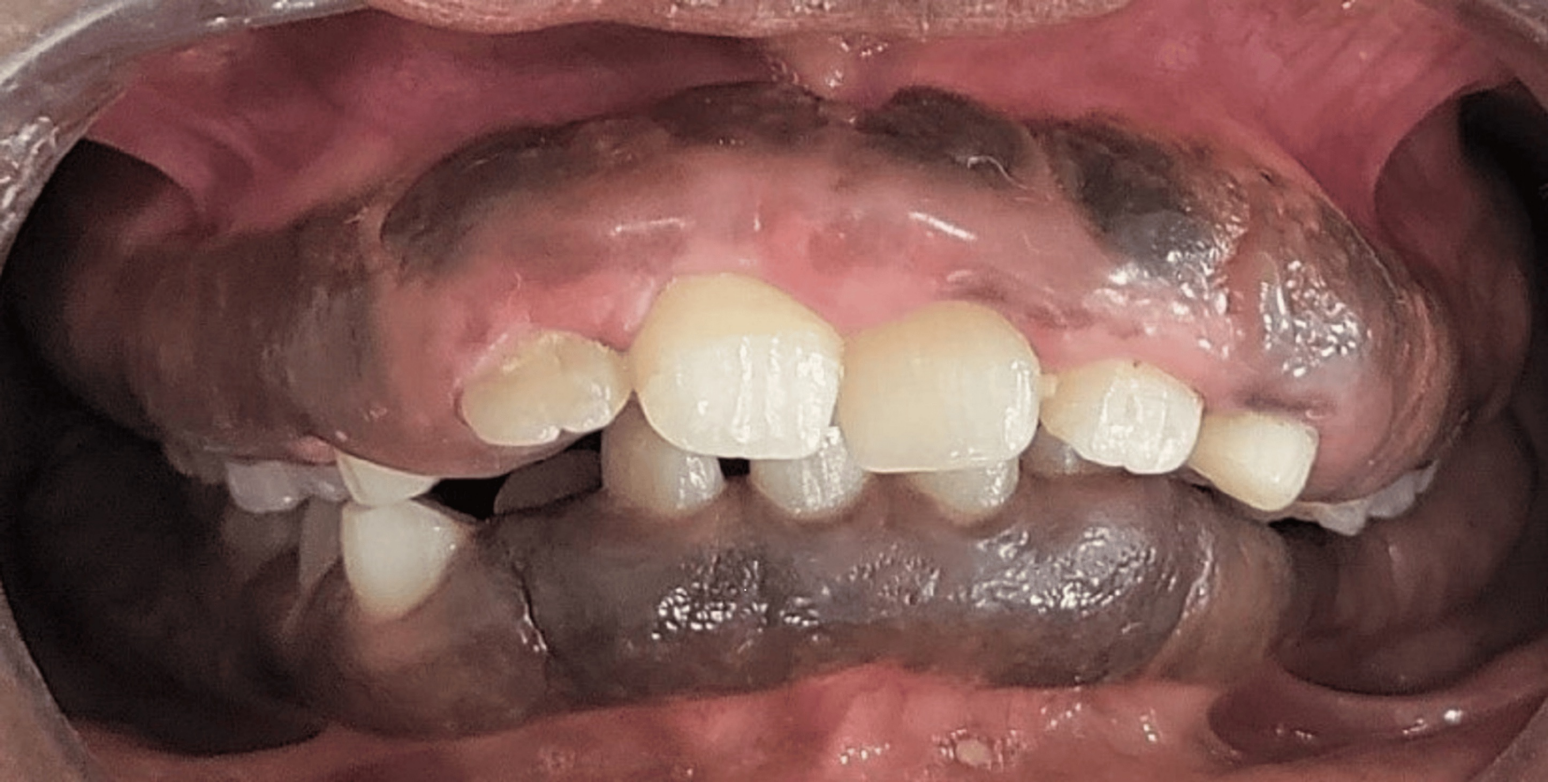
Preoperative frontal view of the generalized diffuse grade III gingival enlargement involving both arches

Extraoral examination revealed incompetent lips and protruded maxilla. Lymph nodes were not palpable and no other TMJ abnormalities were noted. The patient did not give a history of any systemic health issues or drug intake. There was no family history of a similar condition. Intra-oral examination revealed a stage of mixed dentition with generalized massive diffuse (involving marginal gingiva, interdental gingiva, and attached gingiva) grade III GE, i.e., extending up to the occlusal level in the maxillary and mandibular arch. The gingiva was brown, pigmented, fibrotic, and painless occluding the vestibular spaces (Figure 1). Bilaterally in the posterior region, the enlargement was found to reach up to the occlusal level covering the clinical crowns of the teeth. This has definitely compromised the oral hygiene maintenance of the patient.

**Figure 2:**
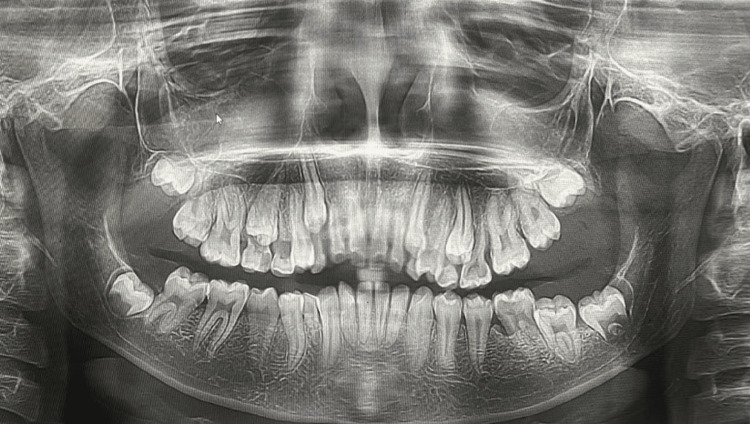
Orthopantomograph depicting a stage of mixed dentition with malocclusion. No significant alveolar changes are seen.

Periodontal examination revealed plaque, subgingival calculus, bleeding on probing, pseudo pockets, halitosis, palatally embedded maxillary premolars in the enlarged gingiva, and grade III mobility in relation to the deciduous maxillary left canine. The orthopantomograph showed a stage of mixed dentition with malocclusion. Many of the primary teeth were seen over-retained in the pantomograph suggesting over-retention due to enlargement of the gingiva eventually causing malocclusion. No significant alveolar changes were seen (Figure 2). A provisional diagnosis of idiopathic GE was given as the family, medical, and drug history was non-contributory. Differential diagnosis of the inflammatory GE was given as malocclusion may have a negative impact on oral hygiene maintenance. Blood investigations were reported to be in the normal range.

After obtaining informed consent, phase I therapy that includes thorough scaling and irrigation was done using saline. Oral hygiene reinforcement was done at every visit. Two percent chlorhexidine mouthwash was prescribed to be used twice a day after toothbrushing. 

**Figure 3:**
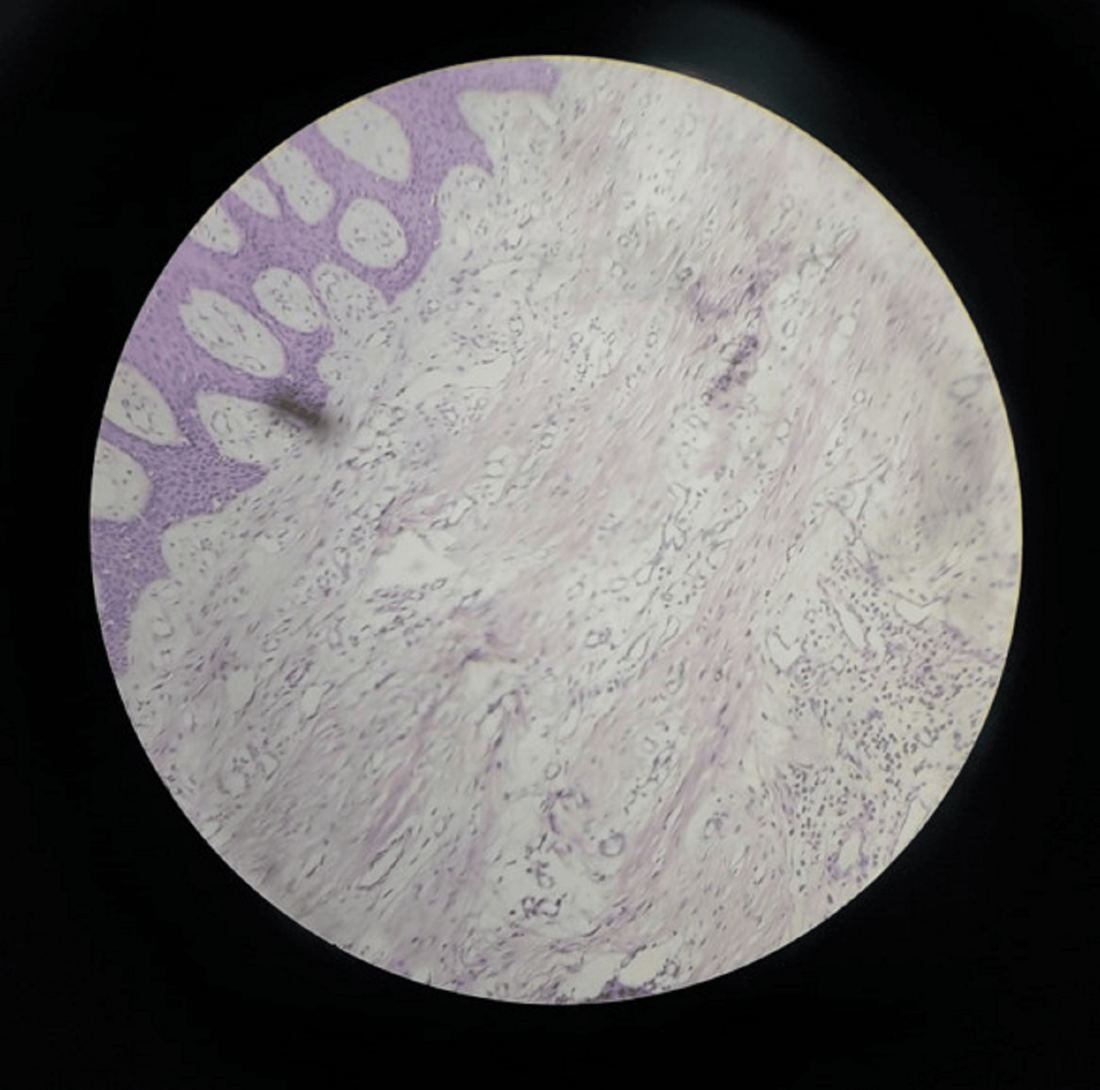
Histopathologic presentation showing a hyperplastic epithelium and abundance of connective tissue with densely populated fibroblasts

After phase I therapy, an incisional biopsy was taken and sent for histopathologic examination, which revealed hyperplastic epithelium, elongated rete pegs, thickened connective tissue, abundance of collagen fibers running in all directions, densely populated fibroblasts, and no blood vessels or inflammatory component suggestive of the fibromatosis (Figure 3). The absence of inflammatory components denotes that malocclusion impairing oral hygiene has not contributed significantly to the enlargement. Based on the clinical, radiographic, and histopathologic findings, the final diagnosis of idiopathic GE was given.

**Figure 4:**
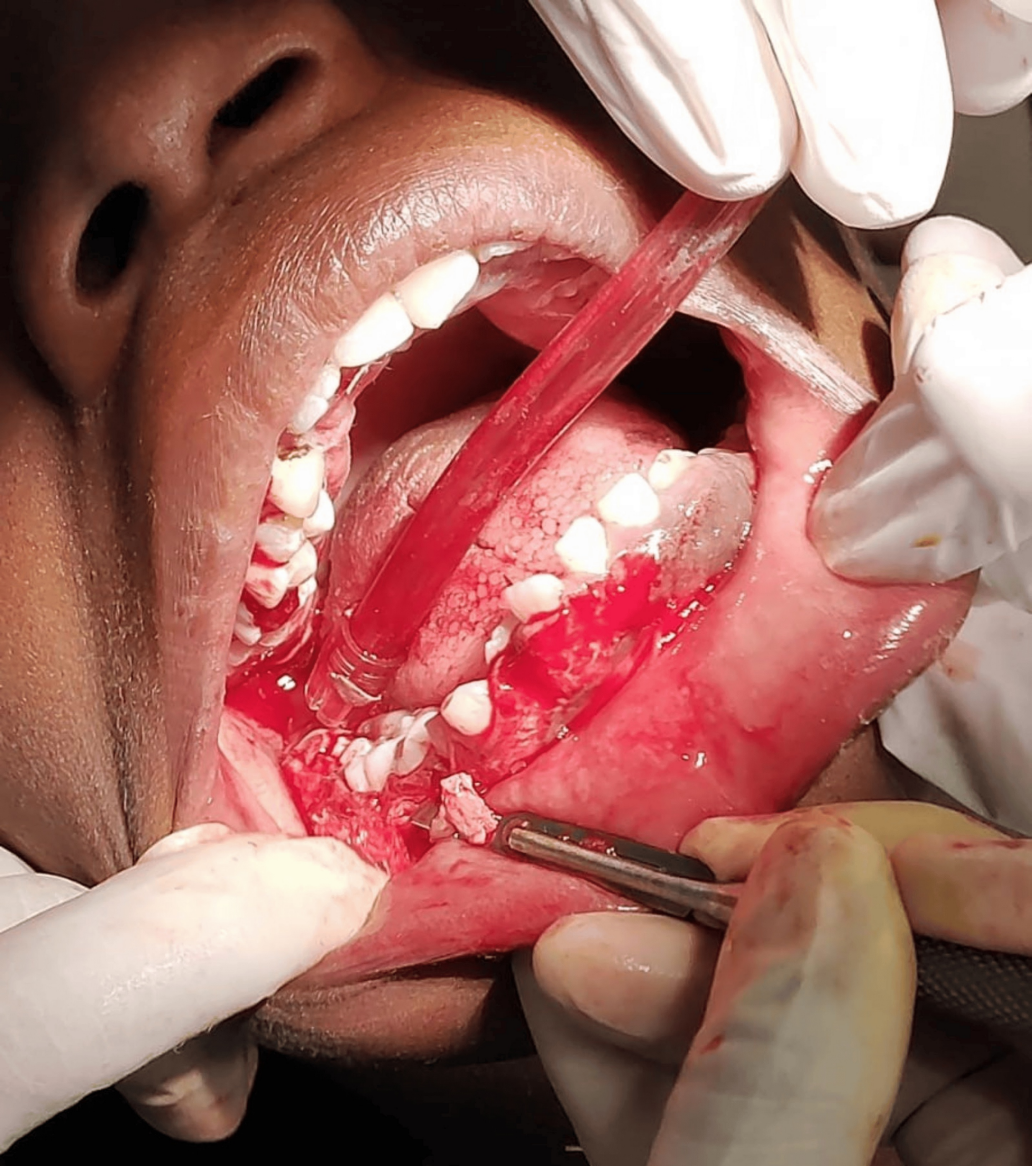
Intraoperative photograph depicting gingivectomy using the conventional technique

**Figure 5:**
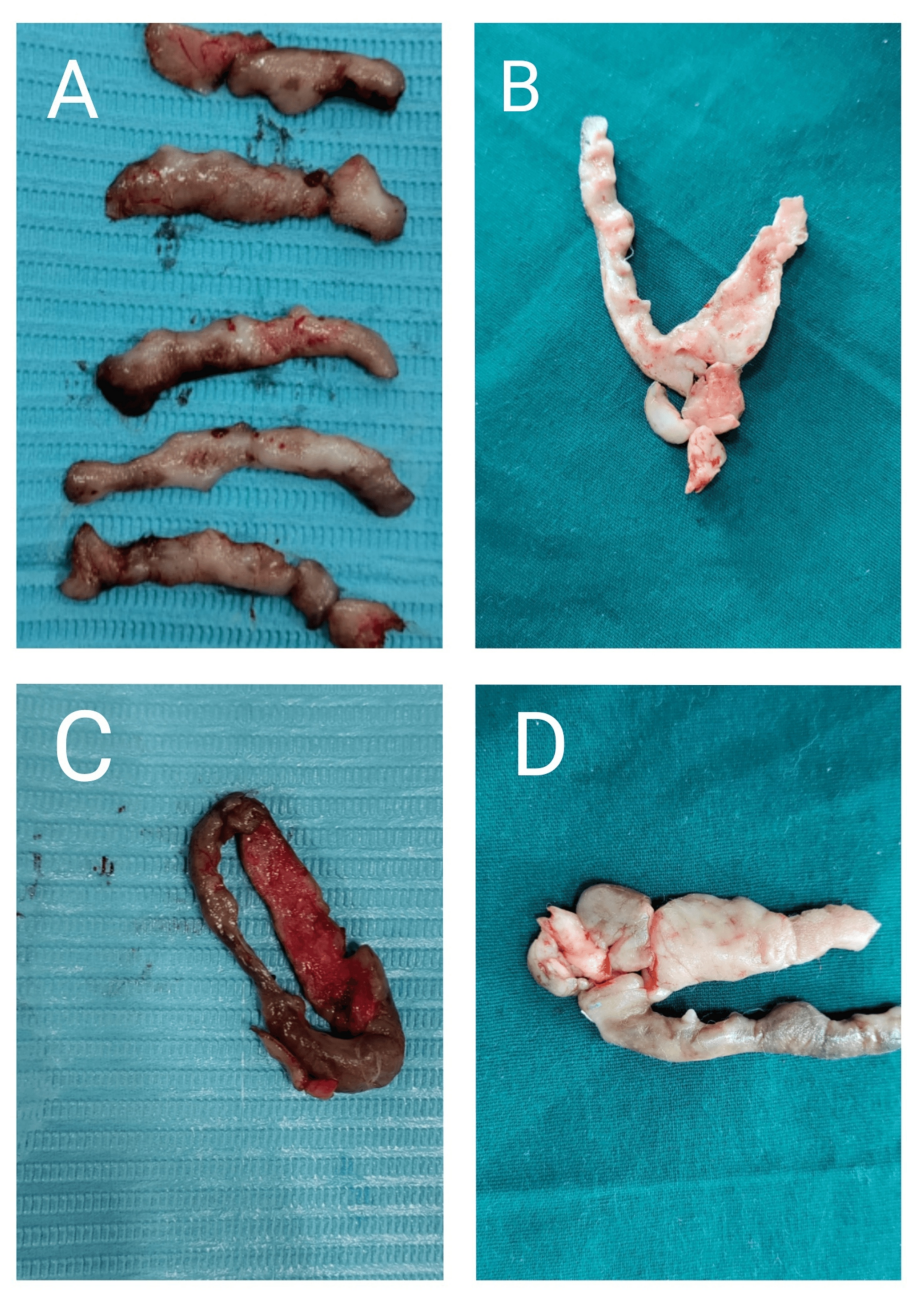
Excised tissue from all the four quadrants (A to D) A) Excised tissue from the first quadrant. B) Excised tissue from the second quadrant. C) Excised tissue from the third quadrant. D) Excised tissue from the fourth quadrant.

Three weeks after the phase I therapy, an external bevel gingivectomy was planned to correct the gingival contour as there was no need for osseous intervention. A conventional technique was planned for the external bevel gingivectomy as the tissue was fibrotic and leathery and this technique would give us precise incisions. It was carried out using number 12 and 15 BP blades, Kirkland and Orban’s knives (Figure 4). A single quadrant was operated at a time. A two-week interval was given before operating the next quadrant. Grade III mobile deciduous maxillary left canine was extracted while operating the respective quadrant. After anesthetizing the quadrant using 2% lignocaine with 1:80,000 adrenaline, the pockets were marked using a pocket marker. Continuous external bevel incisions were given starting from the central incisor on the buccal side and continuing the same on the lingual side. This was followed by sulcular incisions. Multiple thick, leathery, and fibrotic strips of enlarged tissues were excised from all the quadrants. Excision of such extremely leathery continuous long bands of enlarged tissues was made possible due to the precise incisions given using the conventional technique (Figure 5). Such extremely leathery and fibrotic tissue is rare to be found in any type of enlargement cases. Thorough debridement and saline irrigation were done following the excision of the tissue. Gingivoplasty was done using electrocautery to recontour the remaining irregularities. A loop electrode was used in a paint-brush stroke giving 10-15 second cooling intervals. Periodontal dressing was placed at the surgical site for a week to assist healing. Postoperative instructions were given. Analgesics were prescribed. Follow-up after one week, one month, three months, six months, and one year post-surgery was planned.

**Figure 6:**
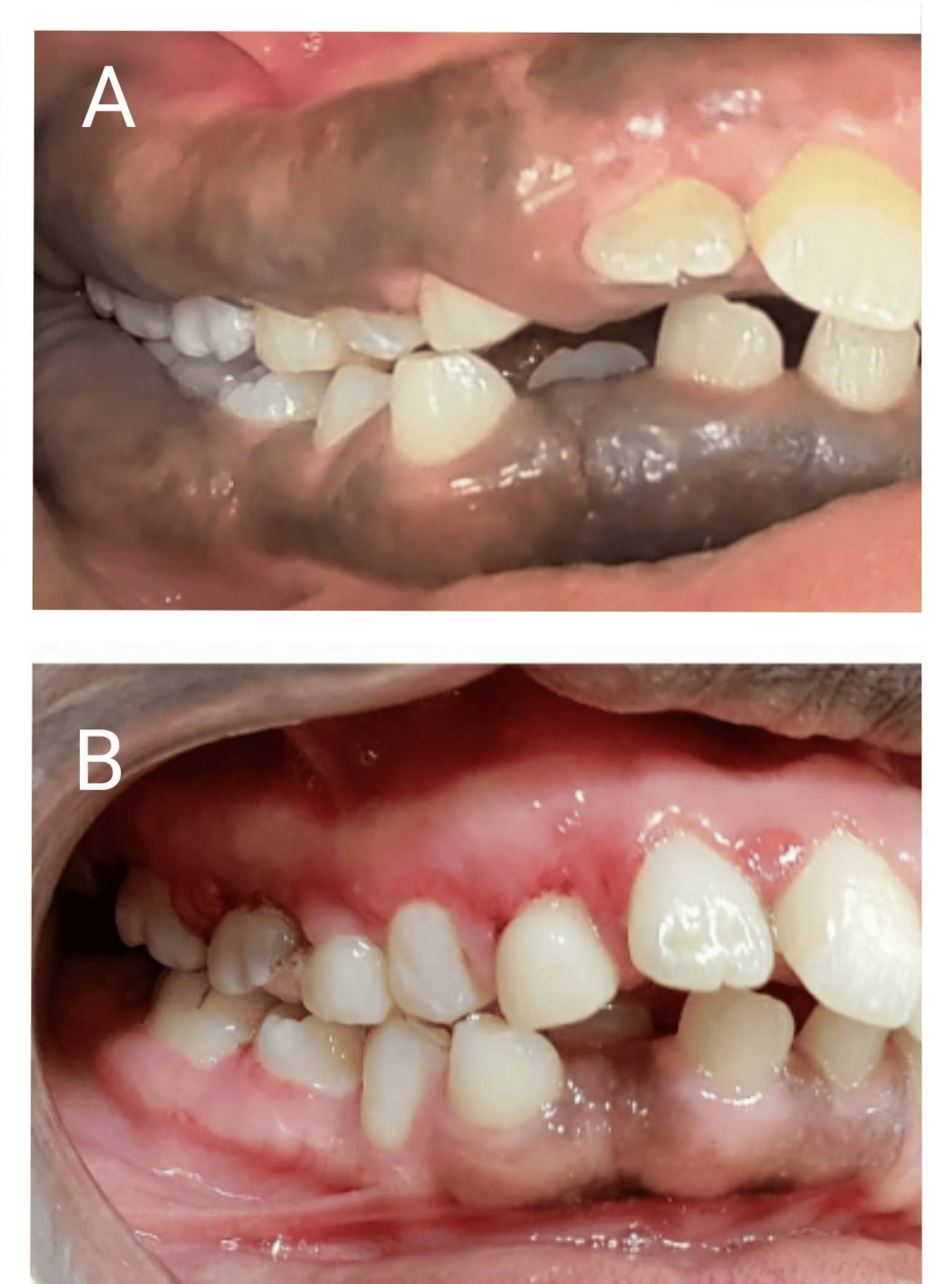
Gingival enlargement on the right side preoperative (A) versus improvement of the gingival status three months postoperative on the right side A: Gingival enlargement on the right side (preoperative). B: Improvement of the gingival status three months postoperative on the right side.

**Figure 7:**
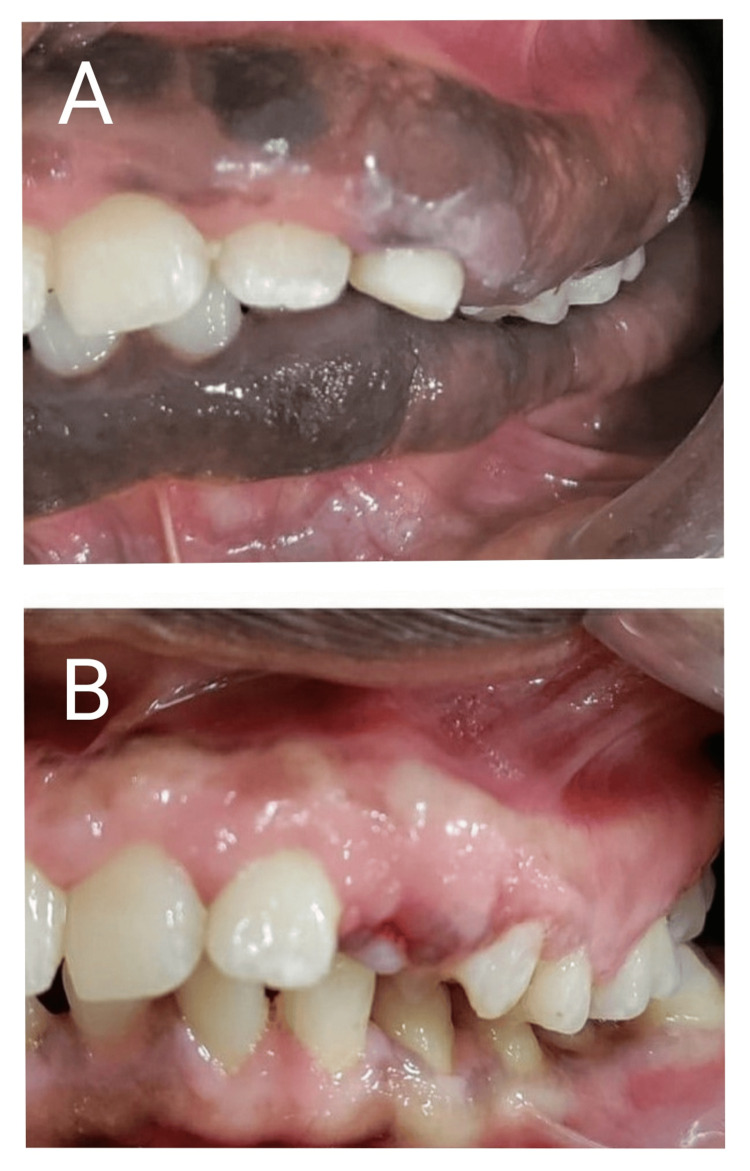
Gingival enlargement on the left side preoperative (A) versus improvement of the gingival status three months postoperative on the left side A: Gingival enlargement on the left side (preoperative). B: Improvement of the gingival status three months postoperative on the left side.

**Figure 8:**
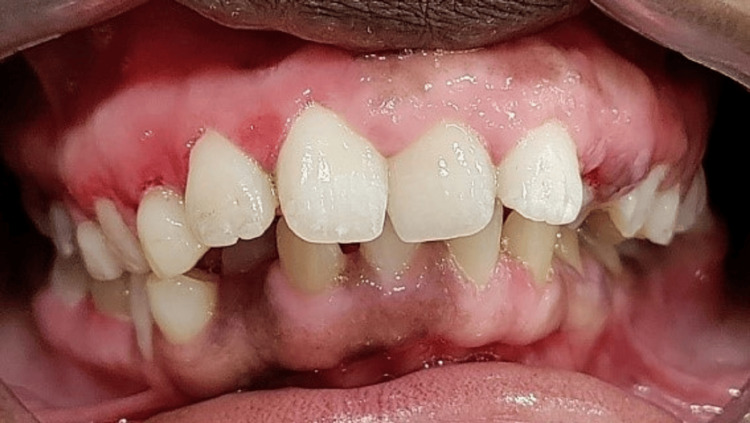
Gingiva restored back to normal in both the arches three months postoperative (frontal view)

**Figure 9:**
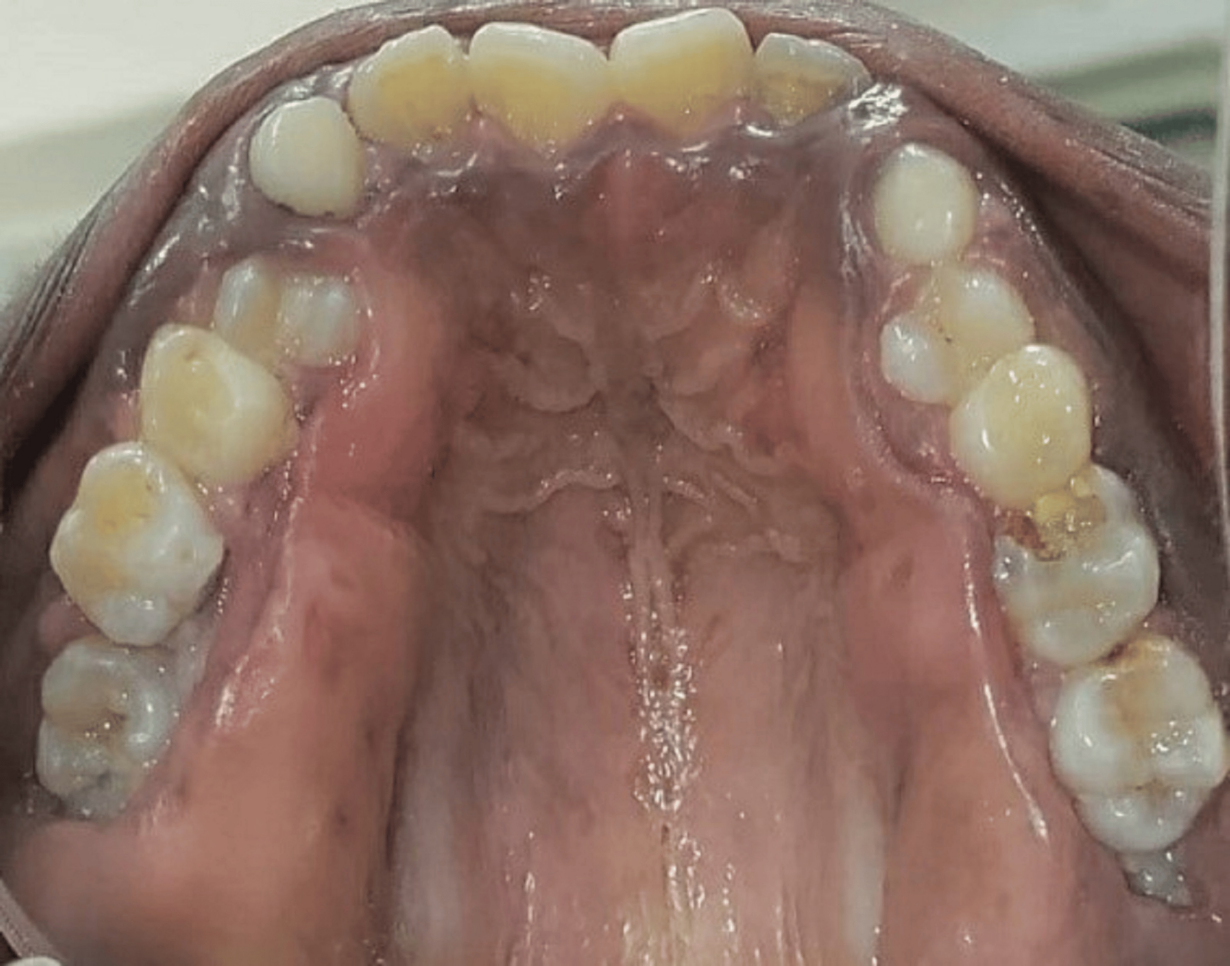
Preoperative palatal view depicting massive enlargement on the palatal side

**Figure 10:**
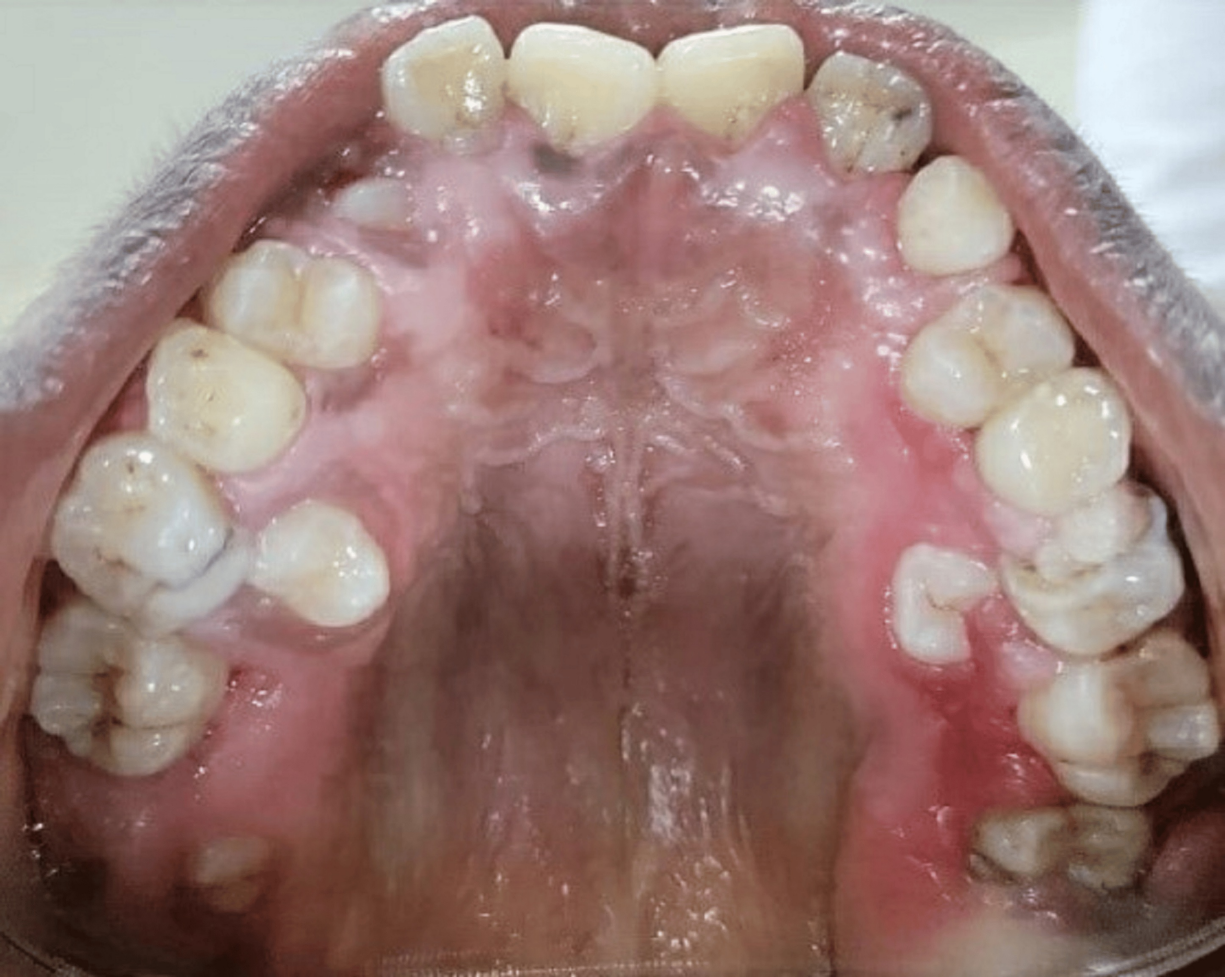
Three months postoperative palatal view exposing the bilaterally embedded pemolars and last molars

**Figure 11:**
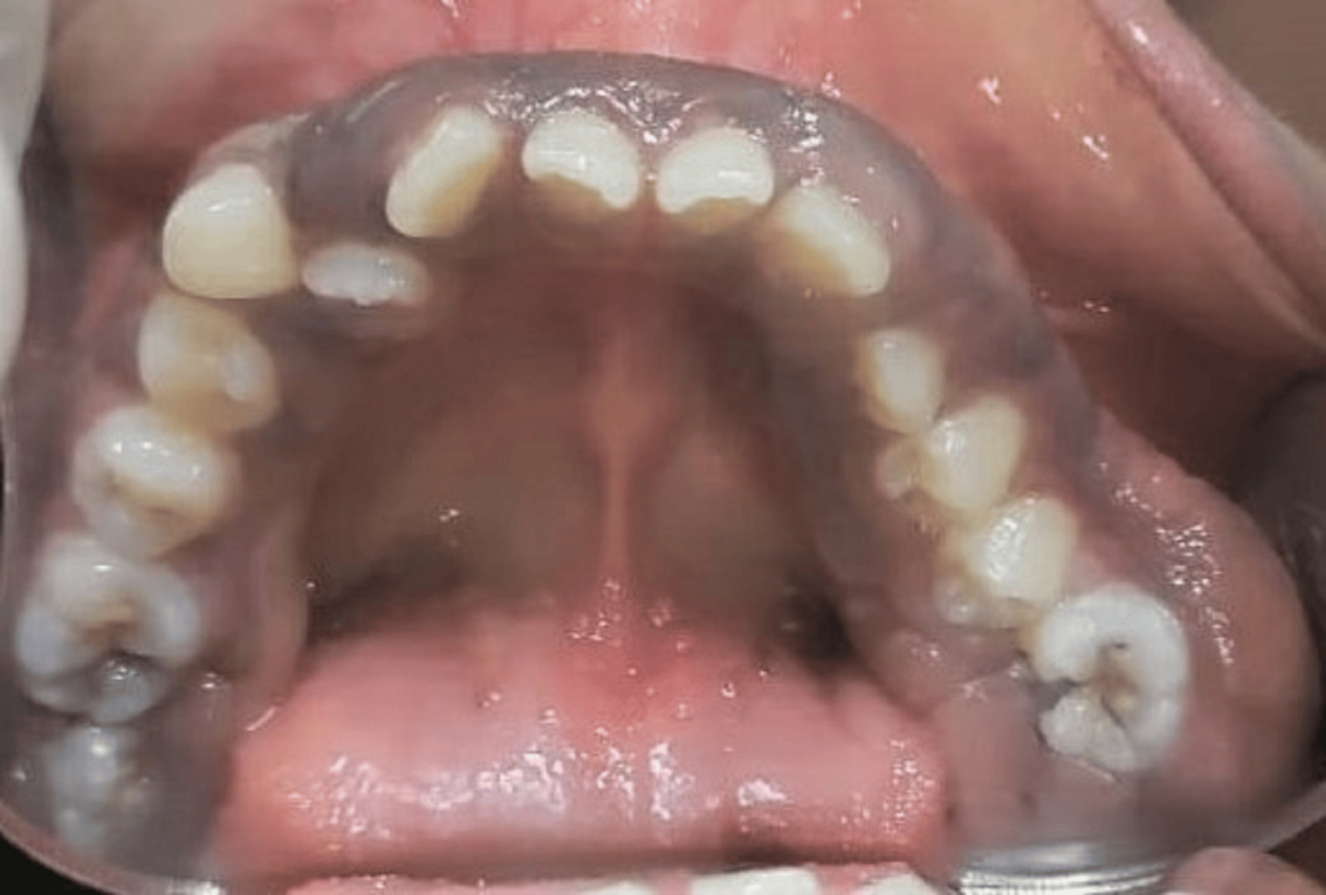
Preoperative occlusal view of the generalized gingival enlargement in the mandibular arch

**Figure 12:**
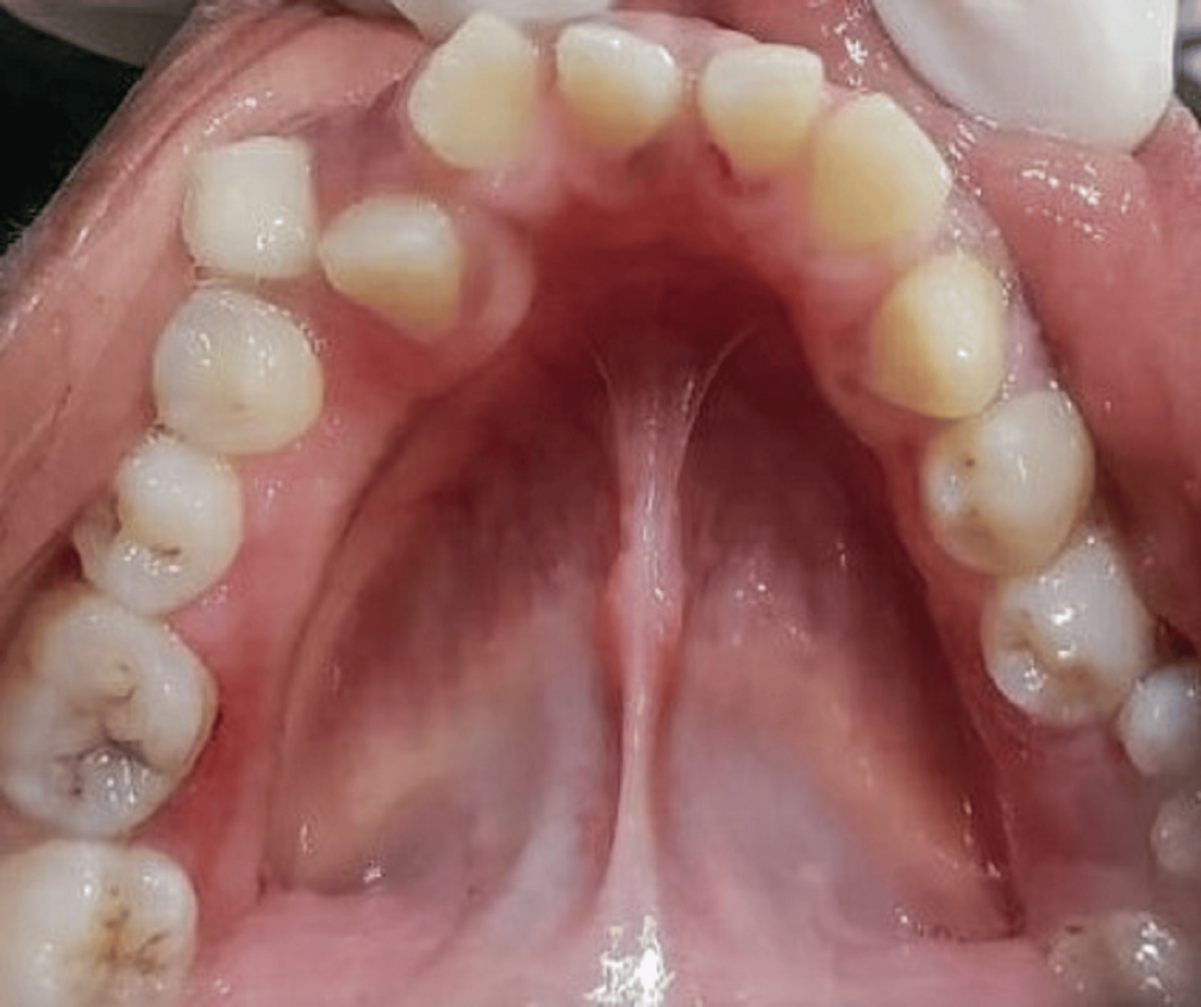
Three months postoperative occlusal view depicting the gingiva in the mandibuar arch restored back to normal

The three-month postoperative evaluation showed that the massive enlargement had subsided with no recurrence and healing was uneventful. All the gingival parameters including the color, contour, consistency, surface texture, position, and bleeding on probing were restored back to normal in all four quadrants. The patient was happy about the simultaneous depigmentation that resulted from the gingivectomy procedure. The vestibular space that was obliterated by the enlarged tissue was restored back to normal (Figures 6-8). The comparison of the occlusal view in the palate preoperative and three months postoperative depicts the significant improvement in the contour of the palatal mucosa. The maxillary premolars and second molars embedded earlier in the enlarged tissue were exposed after gingivectomy. Grade III mobile deciduous maxillary left canine was extracted. After healing of the extraction socket, the underlying erupting permanent canine was seen exposed at the same site (Figures 9, 10). The occlusal view comparison of the gingiva in the mandibular arch preoperative and three months postoperative depicts significant improvement in the contour of the gingiva. The enlarged gingiva had extensively obliterated the buccal vestibule and tongue space (Figures 11, 12). The patient was more compliant with oral hygiene maintenance. The combined surgical and non-surgical approach resulted in good functional and esthetic outcomes. The altered masticatory function, speech, halitosis, difficulty in toothbrushing, and incompetency of lip closure were restored back to normal. The patient’s parents reported a significant improvement in her psychological well-being. Depending on the stability of the gingival status, orthodontic correction of malocclusion would be planned further.

## Discussion

Under the umbrella of gingival and periodontal diseases, GE has gained wide significance. It may be caused by poor oral hygiene, drug intake, leukemia, malignancy, vitamin C deficiency, pregnancy, puberty, or idiopathic. Of all the types, idiopathic enlargement is the rarest variety posing a diagnostic dilemma. Idiopathic GE may be congenital or hereditary. The exact mechanism is not known, but the majority of cases are attributed to hereditary factors, and hence it is comparable to the term hereditary gingival fibromatosis (HGF) [3]. It usually starts with the eruption of deciduous dentition and is rarely present at birth. Sometimes, it starts with the eruption of permanent dentition causing over-retention of deciduous teeth, malocclusion, and arch deformity. It disturbs oral hygiene maintenance, mastication, speech, and esthetics. The extreme consequences are altered swallowing patterns, leading to gastrointestinal disturbances and psychosocial stigma [4].

Idiopathic enlargement is a condition with an unknown cause but presents with a generalized massive diffuse increase in the size of the gingiva as seen in the present case. This can be seen either in isolated form or in association with syndromes, such as Zimmerman-Laband syndrome, Hurler’s syndrome, tuberous sclerosis, Jones syndrome, Cowden syndrome, Niemann-Pick disease, Rutherford syndrome, and Goltz Gorlin syndrome [5,6]. When the medical, physical, drug, and family history is non-contributory, formulating a diagnosis and thereby prognosis poses a challenge to the clinician as seen in the present case. A precise evaluation is essential for organizing the therapy approach and follow-up stage [7].

The interplay between gingival fibroblasts and collagen metabolism decides the fate of GE. The decrease in the activity of collagenase enzyme and over-activity of gingival fibroblast leading to extensive collagen production together result in extensive enlargement of the gingiva [8]. The periodontal fibroblast remains uninvolved in this process, indicating that bone loss caused by enlargement is primarily due to the plaque that has caused inflammation of the periodontium [9]. Based on the underlying cause, the mechanism of enlargement may vary. These include an increase in the proliferation of resident tissue fibroblasts, a reduced level of metalloproteinase synthesis (matrix metalloproteinases MMP-1 and MMP-2), resulting in low levels of extracellular matrix degradation, an increase in collagen type I production, heat-shock protein 47 (hsp47) production, and other extracellular matrix components [10,11].

In the histopathologic examination of the present case, the epithelium appears hyperplastic with elongated rete pegs. There is a marked increase in the amount of connective tissue and shows an abundance of collagen fiber bundles with numerous fibroblasts. The absence of inflammatory components and blood vessels makes the case suggestive of fibromatosis. These findings are congruent with the previous studies [12]. The etiology and pathogenesis of gingival hyperplasia are still not well established; however, it could be directly linked to three factors: individual susceptibility, local factors (dental plaque, caries, and iatrogenic factors), and the action of chemical substances and their metabolites [13].

The course of treatment differs depending on the extent and type of enlargement. It is a common practice to combine surgical and nonsurgical therapies, based on the condition. It is also essential to consider aesthetic and functional requirements [14]. In the present case, a non-surgical therapy aimed at meticulous plaque control was followed by surgical removal of enlarged tissue. The conventional technique was used for gingivectomy to allow precise incisions, low cost of treatment, and early re-epithelialization. This was followed by recontouring the irregularities left at the site using electrocautery. Oral hygiene reinforcement was done at every visit, and hence the patient was well-compliant with plaque control.

The three-month follow-up of the present case reported significant improvement in esthetics, mastication, speech, and oral hygiene maintenance. An extreme change in the patient’s psychology and overall well-being was reported by the patient’s parents. Improved aesthetics due to the overgrowth excision and indirect depigmentation have markedly boosted the self-confidence of the patient. Orthodontic correction of malocclusion would be planned further. This case provides clinical evidence for the successful management of idiopathic GE.

Even though recurrence cannot be predicted, the psychological and functional benefits far outweigh the risk of recurrence. Oral hygiene and the superimposition of plaque accumulation have a crucial effect on the prognosis of GF [15]. The recurrence rate shows conflicting results in the literature [16,17]. This case has to be followed up for the long term to check the recurrence of enlargement and the effect of orthodontic treatment on the gingival and periodontal health.

## Conclusions

A precise case history, clinical examination, and comprehensive treatment plan play a vital role in the management of idiopathic GE. In the present case, as the medical, drug, and family history was non-contributory, the diagnosis of idiopathic GE was given considering the clinical, radiographic, and histopathologic findings. The combined approach of non-surgical and surgical treatment has resulted in good functional and esthetic outcomes. Gingivectomy using conventional technique has resulted in giving precise incisions in extremely fibrotic, leathery, and massive GE. A meticulous plaque control at home and office is reinforced for the long-term success of treatment. Orthodontic correction of malocclusion may be considered depending on the stability of the gingival treatment. Continued follow-up with immediate intervention and patient compliance with oral hygiene maintenance may reduce the recurrence rate.
